# Clinical Outcomes of Cyanoacrylate Closure Versus Radiofrequency Ablation for Saphenous Varicose Veins: A Single-Center Retrospective Study

**DOI:** 10.1155/ijvm/9668464

**Published:** 2025-05-29

**Authors:** Yuki Kamikawa, Takeshi Kinoshita, Yosuke Saito, Tetsuma Oyama, Minoru Tabata, Hirotaka Inaba

**Affiliations:** ^1^Department of Cardiovascular Surgery, Juntendo University Hospital, Tokyo, Japan; ^2^Department of Cardiovascular Surgery, Toda Chuo General Hospital, Saitama, Japan; ^3^Department of Cardiovascular Surgery, Juntendo University Urayasu Hospital, Chiba, Japan; ^4^Department of Cardiovascular Surgery, Juntendo University Shizuoka Hospital, Shizuoka, Japan

**Keywords:** cyanoacrylate closure, phlebitis, radiofrequency ablation, varicose vein, venaseal closure system

## Abstract

**Introduction:** Varicose veins are a common condition affecting millions worldwide. Cyanoacrylate closure (CAC) and radiofrequency ablation (RFA) are widely used minimally invasive treatments. CAC offers advantages such as eliminating tumescent local anesthesia (TLA) and reducing nerve injury risk. However, there are few reports comparing CAC and RFA outcomes in Japan. This study is aimed at evaluating the efficacy and safety of CAC and RFA for treating great saphenous vein (GSV) and small saphenous vein (SSV) varicose veins.

**Materials and Methods:** A retrospective, single-center study was conducted with 157 patients (178 limbs) who underwent either CAC (*n* = 96) or RFA (*n* = 82) from January 2020 to October 2023. Postoperative occlusion rates, complications, and risk factors for phlebitis were analyzed. Follow-up ultrasound examinations were conducted at up to 3 months postoperatively. Statistical analyses included *t*-tests, Mann–Whitney *U* tests, and multivariate logistic regression.

**Results:** Both CAC and RFA achieved a 100% occlusion rate at 3 months. In the CAC group, significantly longer vein segments were treated compared to those in the RFA group (46 ± 14 cm vs. 35 ± 13 cm, *p* < 0.05). However, phlebitis occurred in 15% of the CAC group, whereas none were reported in the RFA group (*p* < 0.05). Multivariate analysis identified preoperative swelling (OR: 5.60, 95% CI: 1.33–23.6, *p* < 0.05) and treated vein length as independent risk factors for phlebitis. All phlebitis cases resolved with conservative treatment. One patient in the RFA group experienced transient paresthesia.

**Conclusion:** CAC is a viable alternative to RFA, demonstrated equivalent occlusion rates, though CAC exhibited a higher incidence of phlebitis. Careful patient selection and perioperative management may help mitigate risks. Further studies with larger cohorts and longer follow-up are needed to optimize treatment protocols and long-term outcomes.

## 1. Introduction

Varicose veins affect approximately 10%–30% of the global population [1, 2]. In Japan, the prevalence among individuals over 40 years old is estimated to be 8.6% (3.8% in male and 11.3% in female), affecting over 10 million people [3].

Treatment options range from conservative measures, such as lifestyle modifications and compression therapy, to invasive treatment for symptomatic cases. Indications for invasive treatment include complications like hyperpigmentation and ulceration, which can impair daily activities. Traditional invasive procedures include vein stripping, high ligation, and sclerotherapy. In Japan, radiofrequency ablation (RFA) has been covered by national insurance since June 2014, and nontumescent therapies for endovascular embolization, such as cyanoacrylate closure (CAC) using the VenaSeal Closure System (Medtronic, Minneapolis, Minnesota, United States), became available since December 2019. RFA, ClosureFast RFA system (Medtronic, Minneapolis, Minnesota, United States), was introduced at our hospital in 2017 and followed by CAC in 2022.

CAC offers several advantages over RFA, including a lower risk of heat-induced nerve injury, the ability to treat longer vein segments, and the elimination of tumescent local anesthesia (TLA). Due to its minimal intraoperative pain, CAC has been adopted as the first-line treatment for approximately 90% of cases at our hospital. Previous studies have demonstrated that CAC is noninferior to RFA, with 3-month closure rates of 99% and 96%, respectively (*p* < 0.01). However, data on CAC outcomes in Japan remain limited, particularly in cases involving the great saphenous vein (GSV) and the small saphenous vein (SSV) [4].

This study is aimed at comparing the postoperative occlusion rates, surgical outcomes, and complications of RFA and CAC as endovascular treatments for GSV or SSV varicose veins.

## 2. Materials and Methods

### 2.1. Ethics Statement

This retrospective, single-center study was approved by our institutional review board (Approval Number: E24-0127), and the requirement for obtaining informed consent was waived due to the retrospective nature of the study. Study information, including its purpose, was posted on the hospital website, providing patients with the opportunity to decline participation.

### 2.2. Study Design

We retrospectively identified 157 patients (totaling 178 limbs) with symptomatic saphenous varicose veins who underwent CAC or RFA at the Department of Cardiovascular Surgery, Juntendo University Urayasu Hospital, between January 2020 and October 2023. Each procedure targeted either the GSV or SSV, with additional stab phlebectomy in cases with branching varicose veins. Anesthesia included local anesthesia and TLA, with optional intravenous anesthesia provided at the patient's request. RFA was the primary treatment until January 2022, after which CAC became the preferred method, except in cases where patients had a preference for RFA or had collagen diseases, including rheumatoid arthritis. After February 2022, there were 10 RFA cases. Cases involving concomitant treatment of both the GSV and SSV were excluded, as intraoperative positional changes were required, leading to inaccurate measurement of operative time. The Clinical, Etiology, Anatomic, and Pathophysiology (CEAP) classification was utilized for the preoperative assessment of varicose vein severity, with the clinical (C) classification categorizing cases as C1 (no visible disease), C2 (varicose veins), C3 (edema), C4 (skin changes), C5 (healing ulcer), and C6 (active ulcer).

### 2.3. Procedures

#### 2.3.1. CAC

In Japan, the VenaSeal Closure System is the only insurance-covered CAC treatment. Since its introduction at our hospital in February 2022, CAC has been the preferred invasive treatment for varicose veins due to its nonreliance on TLA and lower procedural pain. Exceptions include patients with cyanoacrylate allergies, collagen disease, or specific treatment preferences.

The procedure begins with ultrasound (US) mapping of the target vein, followed by a final duplex US assessment in the supine position. Under US guidance, the target vein and its junction with the deep vein, either the saphenofemoral junction (SFJ) or the saphenopopliteal junction (SPJ), are identified. The puncture site is selected at the most distal reflux point, typically near the medial malleolus or a branch point of the saphenous vein. Factors such as vessel diameter, tortuosity, and reflux pattern are considered in the selection process, while nerve proximity is not a primary consideration. The vessel diameter is generally 3 mm or larger, and no cases in this study involved refluxing veins smaller than this threshold.

In cases where the GSV or SSV exhibits mild to moderate tortuosity, the guidewire is advanced through the curved segment. If passage is unsuccessful in isolated segments, a secondary puncture is performed to bypass the affected area, a technique that was used in 10 of 86 cases. In cases with severe, continuous venous tortuosity extending from the proximal to the distal segments, where even a stripping device cannot pass, high ligation and phlebectomy are performed instead of CAC.

A 7-Fr sheath is inserted at the reflux point under local anesthesia. A 0.035-in., 180-cm J-wire guidewire is advanced caudally, followed by the introduction of a 7-Fr introducer and dilator from the VenaSeal system. After the guidewire and dilator are removed, a catheter preloaded with VenaSeal adhesive is inserted through the introducer and advanced to a position 5 cm caudal to the junction under US guidance. Proximal compression of the target vein is achieved using a US probe. Two injections of approximately 0.10 mL of VenaSeal adhesive are administered at 1-cm intervals at the targeted site, followed by 3 min of local compression. Subsequent injections and compressions, performed at 30-s intervals using both the US probe and manual hand pressure, continue until the entire target vein segment has been treated.

Following adhesive application, the sheath and catheter are removed, and compression is applied to the catheter entry site until hemostasis is confirmed. If necessary, additional stab phlebectomy is performed. Duplex US is then used to confirm occlusion of the target vein and to ensure that no thrombus or adhesive migration has occurred. Once these criteria are met, the procedure is concluded. If stab phlebectomy is performed, a self-adhesive bandage is applied.

Postoperatively, patients receive nonsteroidal anti-inflammatory drugs (NSAIDs) for 5 days to manage inflammation and potential allergic reactions related to the adhesive.

#### 2.3.2. RFA

The procedure began with US mapping, followed by a final duplex US assessment in the supine position. Under US guidance, the target vein and its junction were identified. A 7-Fr sheath was inserted 15 cm below the knee for GSV cases or in the midlower leg for SSV cases to minimize the risk of paresthesia from TLA. A ClosureFast catheter was advanced through the sheath and positioned 4 cm distal to the junction, with its tip confirmed via US.

A TLA solution containing sodium bicarbonate, lidocaine, epinephrine, and saline was injected around the vein, creating a perivenous tumescent halo for anesthesia and thermal protection. The vein was then heated to 120°C at 6.5-cm intervals. If generator power did not drop below 10 W in the final 5 s, additional thermal ablation was applied—up to three times centrally and twice peripherally.

Following ablation, the catheter and sheath were removed, and pressure was applied to achieve hemostasis. If necessary, stab phlebectomy was performed. Duplex US was used to confirm vein occlusion and ensure no thrombus at the junction. Compression bandages were applied for 24 h, after which patients transitioned to compression stockings.

### 2.4. Follow-Up

Patients were scheduled for follow-up outpatient visits at 2 days, 1 month, and 3 months postprocedure. During each visit, US examination was performed to confirm the treated vein closure and to evaluate any complications, such as deep venous thrombosis (DVT), endovenous glue-induced thrombosis (EGIT), or endovenous heat-induced thrombosis (EHIT). The assessment of EGIT or EHIT was done by transverse imaging at the junction level in a standing position. The US examination was performed by a registered physician. Recanalization of the treated vein was identified based on the presence of blood flow in the patent segment exceeding 5 cm from the SFJ or SPJ [4–6]. Postoperative DVT has been reported several times with thrombus or cyanoacrylate extension from the SFJ to the common femoral vein or from the SPJ to the popliteal vein. EHIT was classified into four categories (Classes 1–4) according to the classification proposed by Kabnick et al. Consistent with previous studies, EGIT was also classified based on Kabnick's EHIT classification [7–9]. Phlebitis after CAC was defined as the appearance of erythema, swelling, edema, pain, tenderness, and itching symptoms at the site along the treated saphenous vein that occurred within 2 weeks after surgery.

### 2.5. Statistical Analysis

All data were presented as number (percentages) for categorical variables and mean ± standard deviation or median (range) for continuous variables. An independent *t*-test was performed after verifying variance homogeneity with Levene's test. The Mann–Whitney *U* test was employed to compare categorical variables between the two groups. To identify potential risk factors associated with postoperative phlebitis, univariate analyses were initially conducted to compare patients who developed phlebitis with those who did not in the CAC group. Variables with a *p* value of < 0.10 in the univariate analyses were included in a multivariate logistic regression model, along with age and sex, which were included as fixed factors. Adjusted odds ratio (OR) with 95% confidence interval (CI) were calculated to determine independent risk factors. Statistical significance was defined as *p* < 0.05. All statistical analyses were performed using SPSS Version 27 software (IBM, Armonk, New York, United States).

## 3. Results

### 3.1. Characteristics of the Patients

The baseline characteristics of the study participants are shown in Table 1. In total, 157 patients underwent 178 procedures (CAC group: 80 patients and 96 procedures; RFA group: 77 patients and 82 procedures). The mean age at the time of the procedure was similar between groups (CAC: 67.7 ± 13.2 years, RFA: 68.4 ± 12.0 years, *p* = 0.49). Female distribution was similar in both groups (CAC: 59%, RFA: 57%, *p* = 0.98). The distribution of treated veins differed significantly between the groups. GSV was treated more frequently in the RFA group (72% vs. 87%, *p* < 0.05), whereas SSV was more frequently treated in the CAC group (28% vs. 13%, *p* < 0.05).

The CEAP classification also differed between groups. C2 classification was more prevalent in the RFA group (15% vs. 38%, *p* < 0.05), whereas C4 was more prevalent in the CAC group (34% vs. 21%, *p* < 0.05). The proportion of patients classified as C3 was slightly higher in the CAC group (46% vs. 34%), but this difference was not statistically significant (*p* = 0.13).

### 3.2. Operative Outcomes

The operative outcomes are summarized in Table 2. The mean operative time did not differ significantly between CAC and RFA for both GSV (50 ± 19 min vs. 46 ± 21 min, *p* = 0.13) and SSV (44 ± 15 min vs. 42 ± 18 min, *p* = 0.53). The treated vein length was significantly longer in the CAC group when targeting the GSV (46 ± 14 cm vs. 35 ± 13 cm, *p* < 0.05). However, no significant difference was observed for SSV procedures (22 ± 8.0 cm vs. 17 ± 8.8 cm, *p* = 0.14). The number of stab phlebectomies was similar between the groups for either GSV or SSV treatments. At 3 months postoperatively, venous echocardiography confirmed a 100% occlusion rate in both CAC and RFA groups, with no cases of recanalization.

### 3.3. Adverse Events

Adverse events are presented in Table 3. Phlebitis occurred in 14 patients (15%) in the CAC group, whereas no cases were reported in the RFA group (*p* < 0.05). Phlebitis was observed as early as postoperative Day 2 in two patients, while the majority (12 patients) developed symptoms after 1 week. Regarding thrombotic events, EGIT/EHIT Class 2 was observed in one patient (1%) in both groups, but no cases of Class 3 or higher were reported. No patients required anticoagulation therapy. The occlusion rate at 3 months postoperatively was 100% in both CAC and RFA groups. Other complications included paresthesia in one patient (1%) in the RFA group, which resolved after 1 year, and ulcer formation in one patient (1%) in the RFA group. No cases of paresthesia or ulcers were observed in the CAC group.

### 3.4. Risk Factors for Phlebitis

To identify risk factors for postoperative phlebitis, we conducted a comparative analysis between patients with and without postoperative phlebitis in the CAC group (Table 4). Preoperative swelling was significantly more common in the phlebitis group (42.9% vs. 14.6%, *p* < 0.05). Conversely, C3 (edema) was significantly lower (35.7% vs. 47.6%, *p* < 0.05). Treated vein length was longer in the phlebitis group (52.9 ± 11.7 cm vs. 37.2 ± 16.8 cm, *p* < 0.05). They also had a higher injection volume of CA (1.76 ± 0.25 mL vs. 1.34 ± 0.52 mL, *p* < 0.05). No significant associations were found with other clinical characteristics. In the CAC group, significantly longer vein segments were treated compared to those in the RFA group (46 ± 14 cm vs. 35 ± 13 cm, *p* < 0.05; 95% CI: 7.2–16.8). Similarly, among patients with and without phlebitis, the treated vein length was significantly greater in those who developed phlebitis (53 ± 12 cm vs. 37 ± 17 cm, *p* < 0.05; 95% CI: 8.3–24.9). In the multivariate logistic regression analysis (Table 5), preoperative swelling (OR: 5.60, 95% CI: 1.33–23.6, *p* < 0.05) was identified as an independent risk factor for phlebitis.

## 4. Discussion

This study compared the surgical outcomes and postoperative complications of CAC and RFA for the treatment of varicose veins. Both groups achieved excellent occlusion rates, with US confirming a 100% occlusion rate for both the GSV and SSV at 3 months postoperatively. These results are consistent with previous studies, which have demonstrated the efficacy of CAC as comparable to RFA [4].

One key finding was the significant difference in treated vein length for the GSV, which was longer in the CAC group (46 ± 14 cm) compared to the RFA group (35 ± 13 cm, *p* < 0.05). Although the treated vein length for the SSV also tended to be longer in the CAC group (22 ± 8.0 cm vs. 17 ± 8.8 cm), this difference did not reach statistical significance (*p* = 0.14). The longer treated vein length in CAC is attributed to the flexibility of selecting the puncture site at the lowest reflux point. In contrast, RFA puncture sites are limited to below the knee for GSV or midlower leg for SSV to reduce the risk of nerve damage. Longer treated vein lengths are advantageous for occluding refluxing perforator veins, which are known to contribute significantly to recurrence [10]. In this study, RFA was performed on a patient classified as C5 preoperatively in the RFA group, resulting in postoperative ulcer deterioration. This outcome was attributed to residual reflux from a perforating vein below the knee.

The operative time did not differ significantly, although the CAC group tended to have a longer operative time (GSV: 50 ± 19 vs. 46 ± 21, *p* = 0.13, SSV: 44 ± 15 vs. 42 ± 18, *p* = 0.53). A previous study has reported significantly longer operative times for CAC [4], primarily due to the adhesive pressure time required for each injection and the potential influence of the learning curve. At our hospital, the first 96 CAC cases were performed during the early adoption phase after its introduction in February 2022, which may have prolonged the procedure duration. However, recent procedural advancements, including the approval of double- and triple-segment approaches in September 2022, may reduce operative times for CAC in the future.

Phlebitis was observed in 15% of the CAC group. This rate is within the reported range for CAC when using the VenaSeal Closure System (6%–20.8%) [4, 11–14]. Phlebitis is often caused by a Type IV hypersensitivity reaction, foreign body reaction, or superficial venous thrombosis [14, 15], and most symptoms are transient, sometimes without itching or hives. Treatment with NSAIDs or antihistamines is recommended, whereas steroids are recommended in severe cases. In our department, we have been administering anti-inflammatory drugs for 5 days postoperatively while considering renal function. Ten of the 15 patients with postoperative phlebitis in the CAC group were treated with additional antihistamines, and symptoms in all were relieved 1 month after surgery.

Phlebitis was significantly associated with preoperative swelling and showed a trend with treated vein length in bivariate analysis, while multivariate logistic regression confirmed both as independent risk factors. Preoperative swelling, a known marker of venous stasis and inflammation, may predispose patients to an exaggerated inflammatory response after cyanoacrylate injection. The presence of swelling could also indicate pre-existing endothelial activation or damage, which may amplify the local inflammatory reaction caused by the adhesive material. These findings highlight the importance of assessing and managing preoperative swelling, potentially through optimized patient selection or pretreatment anti-inflammatory therapies.

One advantage of CAC is its capability to treat large areas effectively. However, a notable limitation is its potential to increase the risk of phlebitis. These findings suggest the importance of recognizing preoperative swelling and treated vein length when assessing patient risk for phlebitis. Strategies to mitigate this risk may include prophylactic anti-inflammatory medications, optimizing the adhesive volume, establishing preoperative allergy testing protocols for cyanoacrylate, or developing improved adhesive formulations.

Although patient satisfaction and postprocedure pain are critical aspects of varicose vein treatment outcomes, these parameters were not systematically assessed in the present study. Based on clinical experience at our institution, both CAC and RFA were generally well-tolerated, with most patients reporting minimal postprocedural discomfort. However, formal patient-reported outcome measures (PROMs) were not collected. Future prospective studies including a systematic assessment of patient satisfaction and pain levels are warranted to comprehensively evaluate the benefits and drawbacks of these procedures.

This study had some limitations. First, this was a retrospective single-center study with a small sample size. The inability to identify significant factors in the risk analysis for phlebitis may also be attributed to the small sample size, resulting in reduced statistical power. Second, CAC was the first choice of surgery after February 2022. Finally, the CAC group involved the first 96 cases after introducing the technique at our hospital, which may imply learner bias. Future studies that expand this work with longer follow-up periods to assess long-term outcomes and potential complications are needed.

## 5. Conclusions

Both CAC and RFA achieved a 100% occlusion rate at3 months, confirming their comparable efficacy. CAC enabled treatment of longer vein segments but was associated with a 15% incidence of phlebitis, with preoperative swelling and treated vein length identified as risk factors. While CAC offers advantages such as eliminating tumescent anesthesia and reducing nerve injury risk, strategies to minimize complications are needed. Careful patient selection and perioperative management may help mitigate risks. Further studies should evaluate long-term outcomes and refine treatment protocols.

## Figures and Tables

**Table 1:** Baseline clinical data of patients undergoing endovascular treatment for varicose veins (*n* = 157).

**Variable**	**CAC (** **n** = 80**)**	**RFA (** **n** = 77**)**	**p** ** value**
Age (years)	67.7 ± 13.2 (41–88)	68.4 ± 12.0 (30–90)	0.49
Female	47 (59)	44 (57)	0.98
Number of procedures	96	82	
GSV	69 (72)	71 (87)	< 0.05
SSV	27 (28)	11 (13)	< 0.05
Present symptoms			
Pain	18 (19)	20 (24)	0.36
Heaviness	31 (32)	18 (22)	0.19
Swelling	18 (19)	17 (21)	0.74
Cramping	19 (20)	9 (11)	0.17
Itching	6 (6)	7 (9)	0.56
Ulcer	2 (2)	6 (7)	0.09
Others	7 (7)	13 (16)	0.07
CEAP classification			
C2	14 (15)	31 (38)	< 0.05
C3	44 (46)	28 (34)	0.13
C4	33 (34)	17 (21)	< 0.05
C5	3 (3)	1 (1)	0.39
C6	2 (2)	5 (6)	0.17

*Note:* Categorical variables are presented as frequency (percent). Continuous variables are presented as mean ± standard deviation and range.

Abbreviations: CAC, cyanoacrylate closure; CEAP, clinical, etiological, anatomical, pathophysiological; GSV, great saphenous vein; RFA, radiofrequency ablation; SSV, small saphenous vein.

**Table 2:** Endovascular treatment outcomes in patients with varicose veins.

**Variable**	**GSV**			**SSV**		
	**CAC (** **n** = 69**)**	**RFA (** **n** = 71**)**	**p** ** value**	**CAC (** **n** = 27**)**	**RFA (** **n** = 11**)**	**p** ** value**
Operative time (min)	50 ± 19 (18–117)	46 ± 21 (12–131)	0.13	44 ± 15 (20–78)	42 ± 18 (18–84)	0.53
Treated vein length (cm)	46 ± 14 (16–76)	35 ± 13 (6.5–58)	< 0.05	22 ± 8.0 (10–40)	17 ± 8.8 (5–32)	0.14
Stab phlebectomy	4.8 ± 5.3 (0–25)	3.9 ± 4.1 (0–23)	0.61	5.7 ± 5.2 (0–20)	4.9 ± 3.4 (0–13)	0.90
Injection volume of CA (mL)	16 ± 4.2 (0.7–2.5)	—		8.6 ± 2.5 (0.5–1.5)	—	
Closure rate (%)	100	100		100	100	
Complication	21 (30)	3 (4)	< 0.05	1 (4)	1 (9)	0.11

*Note:* Categorical variables are presented as frequency (%). Continuous variables are presented as mean ± standard deviation and range.

Abbreviations: CA, cyanoacrylate; CAC, cyanoacrylate closure; GSV, great saphenous vein; RFA, radiofrequency ablation; SSV, small saphenous vein.

**Table 3:** Adverse events.

**Variable**	**CAC (** **n** = 96**)**	**RFA (** **n** = 82**)**	**p** ** value**
Phlebitis	14 (15)	0	< 0.05
DVT	1 (1)	1 (1)	0.91
EGIT/EHIT Class 2	1 (1)	1 (1)	0.91
EGIT/EHIT Class 3 or 4	0	0	
Paresthesia	0	1 (1)	0.28
Ulcer	0	1 (1)	0.28

*Note:* Categorical variables are presented as frequency (percent).

Abbreviations: CAC, cyanoacrylate closure; DVT, deep venous thrombosis; EGIT, endovenous glue-induced thrombosis; EHIT, endovenous heat-induced thrombosis; RFA, radiofrequency ablation.

**Table 4:** Comparison of clinical characteristics and outcomes between phlebitis and nonphlebitis in the CAC group.

**Variable**	**Phlebitis (** **n** = 14**)**	**Nonphlebitis (** **n** = 82**)**	**p** ** value**
Age	66 ± 16 (30–90)	67 ± 13 (31–87)	0.27
Female	7 (50)	49 (60)	0.46
Present symptoms			
Pain	3 (21)	15 (18)	0.60
Heaviness	4 (29)	27 (33)	0.49
Swelling	6 (43)	12 (15)	< 0.05
Cramping	1 (7)	17 (21)	0.23
Itching	1 (7)	5 (6)	0.77
Ulcer	0	2 (2)	0.23
CEAP classification			
C2	2 (14)	12 (15)	0.95
C3	5 (36)	39 (48)	< 0.05
C4	7 (50)	26 (32)	0.15
C5	0	3 (4)	0.14
C6	0	2 (2)	0.23
Treated GSV	14 (100)	55 (67)	< 0.05
Treated vein length (cm)	53 ± 12 (37–76)	37 ± 17 (10–70)	< 0.05
Injection volume of CA (mL)	1.7 ± 0.3 (1.4–2.1)	1.3 ± 0.5 (0.5–2.5)	< 0.05

*Note:* Categorical variables are presented as frequency (percent). Continuous variables are presented as mean ± standard deviation and range.

Abbreviations: CA, cyanoacrylate; CEAP, clinical, etiological, anatomical, pathophysiological; GSV, great saphenous vein.

**Table 5:** Multivariate analysis of risk factors for phlebitis in the CAC group.

**Variable**	**OR**	**95% CI**	**p** ** value**
Age	0.98	0.93–1.04	0.53
Female	0.44	0.10–2.00	0.29
Swelling (present symptoms)	5.60	1.33–23.6	< 0.05
C3 (CEAP classification)	0.48	0.12–1.89	0.30
Treated vein length	1.08	0.98–1.19	0.12
Injection volume of CA	1.16	0.06–24.4	0.92

Abbreviations: CA, cyanoacrylate; CEAP, clinical, etiological, anatomical, pathophysiological; GSV, great saphenous vein; OR, odds ratio.

## Data Availability

The data that support the findings of this study are available from the corresponding author, H.I., upon reasonable request.
